# Assessing the effect of bovine *MSTN* variants on pre‐mRNA splicing

**DOI:** 10.1002/age.70073

**Published:** 2026-01-30

**Authors:** Nicolas Gaiani, Dominique Rocha, Arnaud Boulling

**Affiliations:** ^1^ Université Paris‐Saclay, INRAE, AgroParisTech, GABI Jouy‐en‐Josas France

**Keywords:** functional analysis, splicing prediction, splicing variant

## Abstract

The myostatin protein is a potent negative regulator of skeletal muscle growth encoded by the *MSTN* gene. *MSTN* loss‐of‐function variants lead to a particular cattle phenotype characterized by an increase in skeletal muscle mass, known as “double muscling” or “double muscled”. However, most of the *MSTN* causal variants that have been linked to this phenotype lack experimental validation. This is the case, for example, for the five missense *MSTN* variants reported to be causal according to the Online Mendelian Inheritance in Animals. RNA splicing plays a major role in regulating gene expression; therefore, exploring the effects of variants on RNA splicing may provide relevant information on their functional impact. Here, we have set up a full‐length gene assay (FLGA) to functionally assess *MSTN* splicing variants, and we have used it to test the five missense variants plus a well‐described deep intronic splicing variant as a positive control. We also evaluated the performances of SpliceAI and Pangolin, two deep learning‐based splice predictors, to identify potential splicing effects of these six variants. Our FLGA system performed well and showed that none of the missense variants has an effect on splicing, unlike the positive control. For each variant, splicing program predictions were perfectly concordant with the effect observed in the FLGA. We have produced a relevant and powerful assay to analyze *MSTN* splicing variants in cattle. SpliceAI and Pangolin may be efficiently used to screen large datasets of *MSTN* variants and sort the best candidates prior to experimental validation using an FLGA.

The myostatin protein is a potent negative regulator of skeletal muscle growth encoded by the *MSTN* gene (Beyer et al., 2013). In cattle, only the canonical *MSTN* transcript has been identified, predominantly expressed in muscle, sperm, and uterus, with minimal or negligible expression observed in other tissues (Liu et al., 2022). Loss‐of‐function (LOF) variants in the *MSTN* gene are known to be responsible for an increase in skeletal muscle mass in different livestock species, including dog, sheep, cattle, and pig (Aiello et al., 2018). This characteristic muscle phenotype linked to *MSTN* alterations is monogenic and recessive and is referred to as “double muscling” or “double muscled” (DM). The first description of an LOF variant responsible for this trait in cattle was the c.821del11 mutation reported in Belgian Blue (Grobet et al., 1997). Several other LOF variants responsible for the DM phenotype have been identified later in additional breeds, including Blonde d'Aquitaine, Charolaise, Limousine, Marchigiana, and Piedmontese (Aiello et al., 2018; Kostusiak et al., 2023).

The functional interpretation of *MSTN* variants is straightforward when they cause a frameshift or truncation in the protein, thereby resulting in the loss of the essential C‐terminal active domain (McPherron et al., 1997; McPherron & Lee, 1997). However, the causality of a genetic variant in the elaboration of a given phenotype is not always obvious. For instance, the functional interpretation of missense variants is challenging. This class of variant may alter the function of proteins, as well as the amount and the sequence of RNA transcripts, but it may also be neutral. Typically, only the effects of amino acid substitutions are investigated to assess the variant's impact on protein function. However, splicing disruption caused by missense variants is relatively common and has been well documented in humans (Soemedi et al., 2017). This possibility should not be overlooked in farm species such as cattle. Understanding how variants impact splicing may help pinpoint causal variants. Identifying the causal variants involved in agronomic traits is important for understanding the underlying molecular mechanisms and also for developing more accurate genomic predictions through the integration of this information into the evaluation models (Liu et al., 2020).

Splicing is the process by which introns are removed from the primary RNA to obtain a mature RNA and it plays a major role in gene expression. We recently explored the role of splicing variants in the elaboration of cattle phenotypes using a massively parallel reporter assay (Charles et al., 2025). We also designed a full‐length gene assay (FLGA) to specifically assess the effect of *DGAT1* variants on pre‐mRNA splicing (Gaiani et al., 2023). Using it, we confirmed the previously described effect of the p.M435L missense variant on the skipping of the *DGAT1* exon 16 (Gaiani et al., 2023; Lehnert et al., 2015). Such assays can provide a better understanding of the functional impact of genetic variants, helping to classify them as causal or not.

Here, we designed an FLGA to analyze putative *MSTN* splicing variants. We used it to address the hypothesis that bovine *MSTN* missense variants responsible for the DM phenotype may decrease the function of the gene via splicing disruption. To retrieve such variants, we browsed the Online Mendelian Inheritance in Animals (OMIA) database (https://omia.org/home/) and found five missense variants assigned to the DM phenotype (OMIA:000683‐9913): p.L64P (Dierks et al., 2015), p.F94L (Grobet et al., 1998), p.S105C (Dunner et al., 2003), p.D182N (Dunner et al., 2003), and p.C313Y (Kambadur et al., 1997). The deep intronic c.748‐799T>G variant, which was described in vivo to create a cryptic exon, was included in our study to serve as a positive control (Bouyer et al., 2014). In addition, we used SpliceAI and Pangolin programs to predict the effect of these six variants on *MSTN* splicing, as we have recently validated them for use in cattle (Charles et al., 2025). This enabled us to assess whether these bioinformatic tools could be useful in classifying and filtering *MSTN* variants before any functional validation by FLGA. This can be relevant if a large number of variants need to be analyzed.

To implement the FLGA, the whole sequence of the bovine *MSTN* gene (ENSEMBL:ENSBTAG00000011808) has been introduced in the pcDNA3.1 expression vector using the In Fusion HD cloning Kit (Takara) to obtain the pcDNA3.1‐MSTN wild‐type (WT) construct (Figure 1a). A Methods section is provided in Appendix S1. The sequence inserted downstream of the CMV promoter began within *MSTN* Exon 1 and ended within Exon 3, spanning both translation start (ATG) and stop (TGA) codons (Figure 1a). Alternative alleles relative to the five missense variants and the deep intronic c.748‐799T>G variant were then introduced into the pcDNA3.1‐MSTN WT construct using the QuikChange II XL Site‐Directed Mutagenesis Kit (Agilent Technologies) to generate six different pcDNA3.1‐MSTN variant constructs (Figure 1b). pcDNA3.1‐MSTN WT and variant constructs were transfected in the HEK293T cells, which is a widely used model to perform splicing functional tests. The choice of this cell line was detailed previously (Charles et al., 2025). Forty‐eight hours after transfection, total RNA was extracted from transfected cells, and reverse transcription‐PCR were performed using PCR primers T7 and P1 to specifically amplify the cDNA originated from *MSTN* mRNA transcribed from the pcDNA3.1‐MSTN WT and variant constructs (Figure 1a). The resulting PCR products were analyzed by gel electrophoresis to check the size of the obtained amplicons. Finally, the same PCR products were purified on columns using the QIAquick PCR Purification Kit (Qiagen) and analyzed by Sanger sequencing.

**FIGURE 1:**
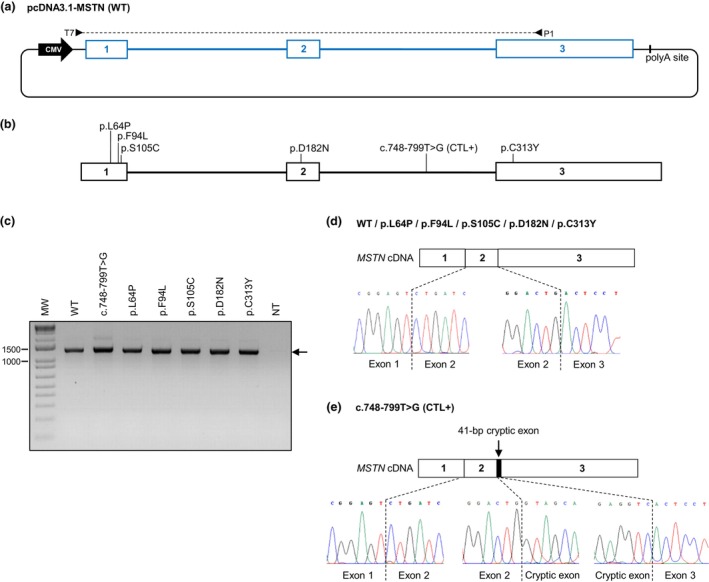
Design of the *MSTN* FLGA and results obtained for five missense variants and controls. (a) Illustration depicting the pcDNA3.1‐MSTN wild‐type (WT) construct. The Blue boxes and lines represent bovine *MSTN* exons and introns, respectively. Black line represents pcDNA3.1 (+) backbone. The T7 and P1 PCR primer pair is indicated, T7 targets pcDNA3.1 5′ UTR and P1 targets *MSTN* exon 3. (b) The five missense variants to be tested and the splicing positive control c.748‐799T>G. (c) Gel electrophoresis of reverse transcription (RT)‐PCR products amplified from HEK293T cells transfected with pcDNA3.1‐MSTN WT and variant constructs. MW, molecular weight; NT, not transfected. (d) Sanger sequencing analysis of WT and missense variants RT‐PCR products performed after on‐column purification. The structure of the cDNA is depicted and the electropherograms are shown at exon–intron boundaries. (e) Sanger sequencing analysis of the c.748‐799T>G RT‐PCR products performed after on‐column purification.

Gel electrophoresis of the reverse transcription‐PCR products showed a single specific amplicon for the c.748‐799T>G variant, as well as for the five missense variants, of a size similar to those obtained with the WT construct (Figure 1c). Sanger sequencing of all of these purified PCR products revealed that the exact sequence of both *MSTN* introns has been fully spliced for each of the five missense variants, as well as for the WT (Figure 1d). This generated a 1317‐bp product that corresponds to the Ensembl canonical *MSTN* transcript (ENSEMBL:ENSBTAT00000015674), leading to the conclusions that: (i) the pcDNA3.1‐MSTN WT construct yielded the expected transcript; and (ii) these missense variants have no effect on splicing. By contrast, the unique mRNA yielded by the c.748‐799T>G variant construct corresponded to a transcript carrying a 41‐bp cryptic exon localized between exons 2 and 3 (Figure 1e). The sequence of this abnormal transcript was identical to that described in vivo by Bouyer et al. (2014) in homozygous carriers of the c.748‐799T>G variant in the Blonde d'Aquitaine breed. Of note, this was the most abundant *MSTN* transcript detected in homozygous c.748‐799T>G animals since the canonical form was barely detectable. This is concordant with the result of our test in which only the transcript that included the 41‐bp cryptic exon has been detected for this variant. If the normal transcript had also been generated, heterozygous positions would have been observed in the electropherogram, due to a mix of normal and abnormal transcripts (Figure 1e).

The five missense variants and the deep intronic c.748‐799T>G variant were analyzed using SpliceAI and Pangolin (Table 1). A score threshold of 0.2 is usually selected to predict splicing variants using both programs (Charles et al., 2025; Jaganathan et al., 2019; Zeng & Li, 2022). Prediction scores obtained for the five missense variants were 0 or 0.01 in any tested conditions, i.e. a gain or a loss of an acceptor or a donor splicing site. This means that according to SpliceAI and Pangolin, they are very unlikely to modify splicing. Scores obtained for the c.748‐799T>G splicing variant validated in vivo were largely above 0.2 with SpliceAI (acceptor gain: 0.73; donor gain: 0.66) and Pangolin (gain: 0.39). Moreover, it is worth noting that if we consider the positions of both new splicing sites predicted by SpliceAI, they were expected to lead to the inclusion of a cryptic exon which exactly corresponded to the 41‐bp cryptic exon observed in vivo and in our FLGA (Table 1).

**TABLE 1:** SpliceAI and Pangolin splicing predictions.

Variant	rsID	Position	Ref	Alt	SpliceAI								Pangolin			
					S_AG	P_AG	S_AL	P_AL	S_DG	P_DG	S_DL	P_DL	S_G	P_G	S_L	P_L
p.L64P	rs449270213	6 279 187	T	C	0	−5	0	15	0	−5	0	−7	0	−5	0	−50
p.F94L	rs110065568	6 279 278	C	A	0	2	0	−32	0	−4	0	10	0.01	2	0	−50
p.S105C	rs5334475047	6 279 310	C	G	0	−20	0	−22	0	22	0	37	0	−22	0	−50
p.D182N	rs5334475067	6 281 368	G	A	0	11	0	1	0	3	0.01	10	0.01	3	0	−50
p.C313Y	rs5334475012	6 283 794	G	A	0	23	0	−1	0	7	0	−1	0	21	0	−50
c.748‐799T>G	rs3423130421	6 282 805	T	G	**0.73**	−41	0	32	**0.66**	−1	0	−50	**0.39**	−41	0	−50

*Note*: Types of effect on splicing: AG, acceptor gain; AL, acceptor loss; DG, donor gain; DL, donor loss; G, gain; L, loss. Interpretation of SpliceAI and Pangolin scores, and how these programs can be used to analyze bovine variants, is explained in detailed in Charles et al. (2025). Scores predicting a splicing effect (≥0.2) are in bold.

Abbreviations: P, position; S, score.

Considering these findings, we can conclude that the five missense variants do not exert their functional effect through a splicing‐related mechanism or else in a minority way, which was not detected by our approach. To our knowledge, no evidence of abnormal splicing was previously reported to be associated with one or more of these variants in vivo, although the bovine muscle transcriptome has been analyzed by RNA‐seq (Guillocheau et al., 2019; Liu et al., 2022). However, abnormal transcripts may be missed in vivo depending on the breeds analyzed and the method used to map RNA‐seq data. To clarify this situation, we wanted to experimentally assess the splicing hypothesis for these five missense variants. Even in the case of negative results, this allows us to focus future functional investigations on other processes involved in gene expression. An impact at the level of the protein function represents a relevant hypothesis to explain why these missense variants are LOF, but no experimental validation has been carried out so far (Johnsson & Jungnickel, 2021). For example, p.L64P was predicted to be deleterious by PolyPhen and SIFT programs based on the physicochemical and evolutionary properties of amino acids (Dierks et al., 2015). Using an approach calculating the impact of missense polymorphisms on the structural and functional characteristics of proteins, the p.C313Y variant was predicted to alter MSTN protein structure and function, whereas the p.F94L variant was not found to clearly affect it (Peka & Balatsky, 2024).

Although we did not highlight any effect of *MSTN* missense variants on splicing, we still have produced and validated a biologically relevant system to functionally analyze variants of this gene. The use of our *MSTN* FLGA may be valuable in validating the causality of a splicing variant in vitro, especially when tissue samples of the carrier animal are not available. In silico screening using SpliceAI and Pangolin programs followed by FLGA represents an efficient tool to search for and validate *MSTN* splicing variants at a large scale. In addition, it enables the functional mechanisms of splicing variants to be explored in depth by testing particular environmental conditions (e.g., exposure to a compound such as cycloheximide) (Zou et al., 2016) or interactions with other variants that may be introduced into the plasmid constructs (Nielsen et al., 2007). Finally, the pcDNA3.1‐MSTN WT and variant constructs we generated may also be used to study the normal or mutated MSTN protein in a cell model since they yield a full transcript carrying the entire *MSTN* coding sequence. The plasmid constructs produced in this study have been deposited in Addgene (see Data Availability Statement).

## AUTHOR CONTRIBUTIONS

A.B.: conceived and designed this study. N.G. and A.B.: managed this study. N.G.: performed laboratory experiments. N.G. and A.B.: analyzed the data. A.B.: writing—original draft preparation. N.G., D.R. and A.B.: writing—review and editing. All authors have read and agreed to the published version of the manuscript.

## CONFLICT OF INTEREST STATEMENT

The authors declare no competing interests.

## Supporting information


Appendix S1.

## Data Availability

All relevant data are available in the manuscript text and Supporting Information. Plasmid constructs may be obtained by contacting www.addgene.org, and have been deposited under following names and Addgene IDs: pcDNA3.1‐MSTN [241319], pcDNA3.1‐MSTN‐L64P [241320], pcDNA3.1‐MSTN‐F94L [241321], pcDNA3.1‐MSTN‐S105C [241322], pcDNA3.1‐MSTN‐D182N [241323], pcDNA3.1‐MSTN‐C313Y [241324], pcDNA3.1‐MSTN‐c.748‐799T>G [241325].
