# Supplementary material for: Assessing the effect of bovine MSTN variants on pre‐mRNA splicing
Source: Anim Genet. 2026 Jan 30;57(1):e70073. doi: 10.1002/age.70073 (PMC12857249; doi:10.1002/age.70073)
Supplement: Supplementary file 1 — Appendix S1. [file AGE-57-0-s001.docx]

**Supplementary Materials**

**Methods**

*Plasmid constructs*

A 6248-bp fragment containing the MSTN genomic sequence from the beginning of the first exon to the three-quarters of the last exon (chr2: 6,278,912-6,285,159) was amplified from a bovine genomic DNA sample. The long-range PCR was performed with 100 ng DNA in a 50 µL reaction mixture containing 25 µL 2X KAPA HiFi HotStart ReadyMix (Roche), and 0.3 μM each primer C1 and C2 (Table S1). The PCR program had an initial denaturation at 95 °C for 3 min, 30 cycles of denaturation at 98 °C for 20 s, annealing at 70 °C for 15 s, extension at 72 °C for 7 min, and a final extension step at 72 °C for 1 min. Amplicons were purified with the QIAquick PCR Purification kit (Qiagen). The pcDNA3.1 (+) expression plasmid was double digested with *Bam*HI and *Xho*I before insertion of the previously generated amplicon by means of the In Fusion HD cloning Kit (Takara), according to the manufacturer’s instructions. The obtained construct carrying the wild-type (WT) bovine *MSTN* gene sequence was named pcDNA3.1-MSTN. It was validated by digestion with AcuI, and the MSTN sequence inserted inside was fully verified by Sanger sequencing using primers T7 and S1 to S13 (Table S1) to be sure that no unwanted mutation was introduced into the construct during the PCR amplification step.

*MSTN* p.L64P (c.191T>C), p.F94L (c.282C>A), p.S105C (c.314C>G), p.D182N (c.544G>A), p.C313Y (c.938G>A) and c.748-799T>G variants were introduced into the pcDNA3.1-MSTN WT construct by means of the QuikChange II XL Site‐Directed Mutagenesis Kit (Agilent Technologies). Mutagenesis was performed in a 51 μl mixture containing 2.5 U PfuUltra HF DNA polymerase, 1 μl dNTP mix, 5 μl 10× reaction buffer, 3 μl QuikSolution, 100 ng pcDNA3.1-MSTN, and 250 ng each forward and reverse mutagenesis primers M1 to M12 (Table S1). The PCR program had an initial denaturation at 95 °C for 1 min, followed by 18 cycles of denaturation at 95 °C for 50 s, annealing at 60 °C for 50 s, and extension at 68 °C for 24 min, and a final extension at 68 °C for 7 min. The post-PCR reaction mixture was treated with *Dpn*I at 37 °C for 1 h then 2 µL of the resulting products were transformed into XL10‐Gold Ultracompetent cells (Agilent Technologies). Transformed cells were spread on LB agar plates with 50 μg/ml ampicillin, and then several selected colonies were used to prepare miniprep plasmid productions with QIAprep Spin Miniprep Kit (including QIAprep 2.0 Spin Miniprep Columns; Qiagen). They were analysed by Sanger sequencing in order to verify the successful introduction of the desired substitution. Constructs harbouring p.L64P, p.F94L, p.S105C, p.D182N, p.C313Y and c.748-799T>G variants were named pcDNA3.1-MSTN-L64P, pcDNA3.1-MSTN-F94L, pcDNA3.1-MSTN-S105C, pcDNA3.1-MSTN-D182N, pcDNA3.1-MSTN-C313Y, and pcDNA3.1-MSTN-c.748-799T>G, respectively. They also may be designated as “pcDNA3.1-MSTN variant constructs” when considered together.

*Cell culture and transfection*

HEK293T cells were cultured in the Dulbecco’s modified Eagle’s medium (DMEM) (Thermo Fisher Scientific) with 10% fetal calf serum (Sigma Aldrich). Twenty-four hours before transfection, 3 × 10^5^ cells were seeded per well in six‐well plates. Then, 1 μg pcDNA3.1-MSTN WT or variant constructs, each mixed with 3 μl Lipofectamine 2000 transfection reagent (Thermo Fisher Scientific), were used for transfection per well.

*RNA extraction and RT-PCR*

Forty‐eight hours after transfection, total RNA was extracted using the RNeasy Mini Kit (Qiagen) according to the manufacturer’s instructions. The RT step was performed with the SuperScript III First-Strand Synthesis System for RT-PCR (Thermo Fisher Scientific) with 1 µg RNA, 2.5 µM Oligo(dT) 20, 500 µM each dNTP, 5 mM MgCl2, 10 mM dithiothreitol, 40 U RNaseOUT and 200 U Superscript III following the manufacturer’s instructions.

The PCR step was performed in a 20 µL reaction mixture containing 0.65 U GoTaq DNA Polymerase (Promega), 1.5 mM MgCl2, 250µM dNTPs, 0.5 µM primer pair T7 and P1 (Table S1), and 1 µL cDNA. The PCR program had an initial denaturation at 95 °C for 2 min, followed by 30 cycles of denaturation at 95 °C for 30 s, annealing at 55 °C for 30 s, extension at 72 °C for 3 min, with a final extension step at 72 °C for 5 min. PCR products were analysed by 1.5% agarose gel electrophoresis.

*Purification of PCR products and Sanger sequencing*

Additionally to gel electrophoresis, PCR products were purified on columns using the QIAquick PCR Purification Kit (Qiagen). Purified products were sent to Eurofins Genomics to be analysed by Sanger sequencing using primers T7 and P1.

*Splicing predictions*

SpliceAI and Pangolin were used to generate splicing predictions for the 6 *MSTN* variants studied here. Specific annotation files were used to run both programs on bovine variants, as previously described in Charles *et al.*, 2025 (reference 8 in the main text).

**Table**

| **Name** | **Sens** | **Sequence** | **Use** |
| --- | --- | --- | --- |
| C1 | F | TACCGAGCTCGGATCCGTTTGGCTTGGCGTTACTCA | *MSTN* Cloning |
| C2 | R | GCCCTCTAGACTCGAGACAGCCATCATGAATCCATAAGT | *MSTN* Cloning |
| T7 | F | TAATACGACTCACTATAGGG | Sequencing of *MSTN* insert |
|  |  |  | RT-PCR |
|  |  |  | Sequencing of RT-PCR products |
| P1 | R | TGTTTGAGGAAGCTATGAACCA | RT-PCR |
|  |  |  | Sequencing of RT-PCR products |
| S1 | F | TCGATGTCCAGAGAGATGCC | Sequencing of *MSTN* insert |
| S2 | F | GGATTGGGAGACAGTATCAGC | Sequencing of *MSTN* insert |
| S3 | F | GAGAGTACCTGGTCTGCACA | Sequencing of *MSTN* insert |
| S4 | F | AGCCTGGCCCTAAAGACAAT | Sequencing of *MSTN* insert |
| S5 | F | TCTCTGGAAAGGAAGTAGGCT | Sequencing of *MSTN* insert |
| S6 | F | CTTGACATGAACCCAGGCAC | Sequencing of *MSTN* insert |
| S7 | F | ATGCTTTCCGTTGATGTGCT | Sequencing of *MSTN* insert |
| S8 | F | GAGCTACTCACTCTTCTGGCT | Sequencing of *MSTN* insert |
| S9 | F | AGTTGGTGACGTGACAGAGG | Sequencing of *MSTN* insert |
| S10 | F | TTCCTCCCTGCTCCTTTCTC | Sequencing of *MSTN* insert |
| S11 | F | AGGCCAATTACTGCTCTGGA | Sequencing of *MSTN* insert |
| S12 | F | GCAATTCTCCTTGTCTTCTGGT | Sequencing of *MSTN* insert |
| S13 | F | GGAGAAGCTGCATTGAAAAGG | Sequencing of *MSTN* insert |
| M1 | F | CAAATCCTCAGTAAACCTCGCCTGGAAACAGC | p.L64P mutagenesis |
| M2 | R | GCTGTTTCCAGGCGAGGTTTACTGAGGATTTG | p.L64P mutagenesis |
| M3 | F | GAACTGATTGATCAGTTAGATGTCCAGAGAGATG | p.F94L mutagenesis |
| M4 | R | CATCTCTCTGGACATCTAACTGATCAATCAGTTC | p.F94L mutagenesis |
| M5 | F | CAGCAGTGACGGCTGCTTGGAAGACGATG | p.S105C mutagenesis |
| M6 | R | CATCGTCTTCCAAGCAGCCGTCACTGCTG | p.S105C mutagenesis |
| M7 | F | CTCATCAAACCCATGAAAAACGGTACAAGGTATACTG | p.D182N mutagenesis |
| M8 | R | CAGTATACCTTGTACCGTTTTTCATGGGTTTGATGAG | p.D182N mutagenesis |
| M9 | F | GCCAATTACTGCTCTGGAGAATATGAATTTGTATTTTTGC | p.C313Y mutagenesis |
| M10 | R | GCAAAAATACAAATTCATATTCTCCAGAGCAGTAATTGGC | p.C313Y mutagenesis |
| M11 | F | GTTGGTGACGTGACAGAGGTCGTAAGTATTATTAAACAGATG | c.748-799T>G mutagenesis |
| M12 | R | CATCTGTTTAATAATACTTACGACCTCTGTCACGTCACCAAC | c.748-799T>G mutagenesis |

**Table S1. Description of PCR and sequencing primers.**
